# Gestational Trophoblastic Disease and Its Outcome at Shri Maharaja Gulab Singh Hospital, Jammu, India: A Retrospective Data Analysis

**DOI:** 10.7759/cureus.116166

**Published:** 2026-09-13

**Authors:** Deepika Sharma, Shreya Verma, Gagandeep Singh

**Affiliations:** 1 Obstetrics and Gynaecology, Government Medical College, Jammu, Jammu, IND

**Keywords:** choriocarcinoma, gestational trophoblastic disease, hydatidiform mole, trophoblastic tumor, β-hcg

## Abstract

Background: Gestational trophoblastic disease (GTD) is a term used to describe a range of benign and malignant trophoblastic diseases, which have a variable clinical presentation and outcome. Early diagnosis via ultrasonography, histopathology, and serial monitoring of β-human chorionic gonadotropin (β-hCG) is crucial to ensure timely treatment and avoid disease progression. The objective of this study was to assess the clinical profile, spectrum of diagnosis, and outcome of GTD in a tertiary care center.

Materials and methods: This is a retrospective observational study carried out in the Department of Obstetrics and Gynecology, Shri Maharaja Gulab Singh (SMGS) Hospital, Jammu, from May 1, 2026, to June 30, 2026. Medical records of 69 patients with a diagnosis of GTD were examined. SPSS version 22.0 (IBM Corp., Armonk, NY) software was used for the analysis of demographic parameters, clinical presentation, histopathological and ultrasonographic findings, pre-evacuation β-hCG level, treatment, and outcome.

Results: Among 69 patients, 38 (55.1%) were aged 25-30 years, 54 (78.3%) had parity ≥2, and 41 (59.4%) had no formal education. Hydatidiform mole was the most common diagnosis, occurring in 65 (94.2%) cases, while choriocarcinoma and invasive mole accounted for 3 (4.3%) and 1 (1.4%) cases, respectively. Passage of grape-like vesicles (27 (39.1%)) and vaginal bleeding (26 (37.7%)) were the predominant clinical presentations. Complete mole was confirmed histopathologically in 60 (87.0%) patients, and 60 (87.0%) had pre-evacuation serum β-hCG levels above 100,000 mIU/mL. Complete resolution was achieved in 62 (89.9%) patients, whereas 7 (10.1%) developed persistent or invasive disease.

Conclusions: The most common type of GTD was hydatidiform mole, where the majority had a high level of β-hCG and a classic clinical picture. The clinical outcome was excellent with early diagnosis, early treatment, and regular β-hCG monitoring, highlighting the need for prompt diagnosis and appropriate surveillance of GTD.

## Introduction

Gestational trophoblastic disease (GTD) is a collection of pregnancy-related disorders caused by abnormal growth of trophoblastic tissue. There are pre-malignant lesions (complete hydatidiform mole (CHM) and partial hydatidiform mole (PHM)) and malignant forms of the disease (invasive mole, choriocarcinoma, placental site trophoblastic tumor (PSTT), and epithelioid trophoblastic tumor (ETT)), which are known as gestational trophoblastic neoplasia (GTN). In recent years, another entity called atypical placental site nodule (APSN) has also been described in the GTD spectrum. Most forms of GTD are associated with the secretion of human chorionic gonadotropin (β-hCG), a highly sensitive biomarker for diagnosis, assessment of response to treatment, and post-treatment surveillance. If β-hCG levels do not decrease or if they increase, one can suspect further evolution to GTN, which happens in 13%-20% of complete moles and 0.5%-5% of partial moles. The prognosis has improved significantly over the last few years due to improvements in β-hCG monitoring, early diagnosis, and chemotherapy, and cure rates have approached 100% for appropriately managed patients [1-3].

GTD is a rare condition, but it shows significant geographic variation. The incidence of hydatidiform mole varies from about 11.5 per 1,000 deliveries in Indonesia to less than 1 per 1,000 deliveries in the United States [4]. Teen pregnancy and women over 35 years of age are important risk factors, with those in the former group having almost twice the risk of developing a molar pregnancy as women aged 20-35 years [5].

Abnormal fertilization and chromosomal imbalance are closely linked to the pathogenesis of GTD. Histopathological examination is the criterion standard method for diagnosis; ancillary techniques, such as short tandem repeat (STR) genotyping and the use of p57 immunohistochemistry, have improved the reliability of complete mole versus partial mole and non-molar gestation diagnoses. There are histological and immunohistochemical differences between the different types of malignant trophoblastic tumors (invasive mole, choriocarcinoma, PSTT, and ETT) and different degrees of biological activity.

Pathological classification is critical as management, prognosis, and follow-up are very different among these entities [6-9].

Since the advent of the widespread use of first-trimester ultrasonography and quantitative estimation of serum β-hCG, the clinical presentation and workup of GTD have significantly changed. In the past, patients complained of classic symptoms, such as excessive bleeding, hyperemesis, early-onset pre-eclampsia, hyperthyroidism, and passage of grape-sized vesicles, but most cases are now detected before symptoms of the complications arise. Ultrasonography plays an important role in the diagnosis, and complete moles usually have a diffuse "snowstorm" appearance, whereas partial moles may have fetal tissue with focal cystic changes in the placenta. There is no doubt that subsequent β-hCG testing is extremely important after uterine evacuation to identify persistent GTN based on the International Federation of Gynecology and Obstetrics (FIGO) guidelines [10]. Although the diagnosis and management of GTD has improved considerably over the last few years, it remains a challenging clinical problem due to its multifaceted nature, its ability to transform into malignancy, and the necessity for long-term follow-up.

Hence, the present retrospective study was conducted to assess the clinical profile, diagnostic spectrum, management, and treatment outcomes of patients with gestational trophoblastic disease at Shri Maharaja Gulab Singh (SMGS) Hospital, Jammu, with a view to adding regional data that may aid in early diagnosis and better patient care.

## Materials and methods

Study design and setting

This hospital-based, retrospective observational study was conducted in the Department of Obstetrics and Gynecology, Shri Maharaja Gulab Singh (SMGS) Hospital, Government Medical College, Jammu. SMGS Hospital is a tertiary care referral center providing specialized obstetric and gynecological services to patients from Jammu and the surrounding regions.

Study duration

This was a retrospective study that included patients diagnosed and managed for gestational trophoblastic disease (GTD) during the one-year period from May 1, 2025, to April 30, 2026. Data extraction, verification, statistical analysis, and preparation of the study report were subsequently undertaken after obtaining approval from the Institutional Ethics Committee from May 1, 2026, to June 30, 2026.

Ethical approval

The research protocol was reviewed by the Institutional Ethics Committee of Government Medical College, Jammu, Sub-Committee, and was “Approved Provisionally” as per the approval issued vide number IEC/GMCJ/2026/2801, dated April 18, 2026. The project was classified under Category C (Cat-C) according to the applicable Standard Operating Procedures and Indian Council of Medical Research guidelines and was registered under registration number C-684.

As the study was retrospective and involved the review of previously recorded hospital data, no direct intervention or contact with the patients was undertaken. Patient identifiers were removed during data collection, and confidentiality was maintained throughout the study. The data were used exclusively for research purposes. The requirement for individual informed consent was waived subject to the conditions specified by the Institutional Ethics Committee.

Study population

The study population consisted of all women diagnosed with gestational trophoblastic disease and treated or evaluated in the Department of Obstetrics and Gynecology, SMGS Hospital, Jammu, during the specified study period. The spectrum of GTD included complete hydatidiform mole, partial hydatidiform mole, invasive mole, choriocarcinoma, placental site trophoblastic tumor, and epithelioid trophoblastic tumor.

Sampling technique

A total enumeration or consecutive sampling technique was employed. All available medical records of women diagnosed with GTD during the study period were screened for eligibility. Every patient fulfilling the predefined inclusion criteria and having adequate clinical information was included. No random sampling was performed because GTD is a relatively uncommon condition and the study intended to include all eligible cases encountered during the study period.

Sample size calculation

The minimum sample size was estimated using the single-population proportion formula: n = Z²pq/d², where n represents the required sample size, Z is the standard normal deviate at a 95% confidence level (1.96), p is the expected proportion, q is 1-p, and d is the allowable absolute error. In the absence of a reliable local estimate, an expected proportion of 50% was considered to provide the maximum required sample size. With an absolute precision of 12%, the calculated sample size was as follows: n = (1.96)² × 0.50 × 0.50/(0.12)² = 66.7.

The calculated sample size was rounded to 67. After allowing approximately 3% for incomplete, unavailable, or unusable medical records, the required sample size was increased to 69. During the study period, 69 eligible patients with adequate records were identified and included in the final analysis.

Inclusion criteria

Women who were diagnosed with any type of gestational trophoblastic disease during the one-year period from May 1, 2025, to April 30, 2026, confirmed by ultrasonography, histopathological examination, serum β-human chorionic gonadotropin (β-hCG) examination, or a combination of these examinations, were included in the study.

Exclusion criteria

Women who suffered intrauterine pregnancies with no complications or obstetric and gynecological conditions that did not fit into the GTD spectrum were not included. Those who had suspected GTD and did not have radiological, biochemical, or histopathological confirmation of the diagnosis were excluded.

Records that were duplicated, patients outside of the specified study window, or records with significant missing data for diagnosis, treatment, or clinical outcome were not included in the final analysis.

Methodology

The necessary information was retrieved from the Medical Records Department, operation theater registers, labor room registers, ward admission/discharge registers, pathology records, radiology records, hospital census records, and available follow-up records. A structured data collection form was pre-designed, and data were entered into this form.

The following variables were recorded: maternal age, residence, socioeconomic status, parity, gestational age at diagnosis, clinical features, history of previous molar pregnancy, uterine size (relative to GA), hemoglobin level, serum beta-hCG level, thyroid function, ultrasound findings, histopathology diagnosis, type of GTD, treatment modality, chemotherapy requirement, complications, length of hospitalization, follow-up status, and clinical outcome.

The treatment modalities evaluated were as follows: suction evacuation, medical management (where applicable), hysterectomy, chemotherapy, supportive treatment, and follow-up with serial serum beta-human chorionic gonadotropin levels. The clinical outcomes were as follows: remission, persistent trophoblastic disease, development of gestational trophoblastic neoplasia, need for further treatment, treatment-related complications, loss to follow-up, and death.

Clinical and demographic variables evaluated

Clinical and demographic variables evaluated included maternal age, parity, gestational age at diagnosis, socioeconomic status, previous molar pregnancy, baseline serum β-human chorionic gonadotropin (β-hCG) level, uterine size, anemia, thyroid dysfunction, GTD subtype, presence of metastatic disease, treatment modality, and follow-up status. No multivariable regression analysis was performed because of the small number of outcome events and the sparse distribution of non-molar GTD cases.

Statistical analysis

Data were entered into a Microsoft Excel spreadsheet (Microsoft Corp., Redmond, WA) and analyzed using IBM SPSS Statistics for Windows version 22.0 (IBM Corp., Armonk, NY). Categorical variables were summarized as frequencies and percentages. Associations between categorical variables were assessed using exact tests when appropriate because several contingency table cells had small expected frequencies. For comparisons of GTD subtype with age group and clinical presentation, the Fisher-Freeman-Halton exact test was used. No correlation or multivariable regression analyses were performed. All statistical tests were two-tailed, and a p-value <0.05 was considered statistically significant.

## Results

A total of 69 patients with gestational trophoblastic disease were included in the study.

As shown in Table 1, the majority of patients belonged to the 25-30-year age group, accounting for 38 (55.07%) cases, followed by 26 (37.68%) patients aged 20-25 years and 5 (7.25%) aged >30 years. No patient was younger than 20 years. Regarding parity, 54 (78.26%) patients had a parity of ≥2, whereas 15 (21.74%) had a parity of <2. Most patients (41 (59.42%)) had no formal education, while 14 (20.29%) had primary education, 11 (15.94%) had secondary education, and only 3 (4.35%) had tertiary education. Housewives constituted 38 (55.07%) of the study population, while 31 (44.93%) were working women. With respect to religion, 43 (62.32%) patients were Muslim, 19 (27.54%) were Hindu, and 7 (10.14%) belonged to other religious groups.

**Table 1:** Sociodemographic Characteristics of the Study Participants (N = 69)

Variable	Category	Number	%
Age group (years)	<20	0	0.00
	20-25	26	37.68
	25-30	38	55.07
	>30	5	7.25
Parity	<2	15	21.74
	≥2	54	78.26
Education	No formal education	41	59.42
	Primary	14	20.29
	Secondary	11	15.94
	Tertiary	3	4.35
Occupation	Housewife	38	55.07
	Working	31	44.93
Religion	Hindu	19	27.54
	Muslim	43	62.32
	Others	7	10.14
Total		69	100.00

Table 2 demonstrates the distribution of GTD subtypes according to age group. Hydatidiform mole was the most common subtype overall, being observed in 65 (94.20%) patients, whereas choriocarcinoma was present in 3 (4.35%) and invasive mole in 1 (1.45%) patient. Among women aged 20-25 years, 23 of 26 (88.46%) had hydatidiform mole and 3 (11.54%) had choriocarcinoma. Among those aged 25-30 years, 37 of 38 (97.37%) had hydatidiform mole, while 1 (2.63%) had invasive mole. All five patients aged >30 years had hydatidiform mole. No patient was recorded in the <20-year age group. The Fisher-Freeman-Halton exact test showed no statistically significant association between age group and GTD subtype (p=0.186).

**Table 2:** Distribution of Gestational Trophoblastic Disease Subtypes According to Age Group (N = 69) Fisher-Freeman-Halton exact test: p = 0.186 The <20-year age group contained no participants and was not included in the statistical comparison. Percentages within individual age groups are row percentages; percentages in the total column represent proportions of the total study population.

Age group (years)	Hydatidiform mole, number (%)	Invasive mole, number (%)	Choriocarcinoma, number (%)	Total, number (%)
<20	0 (0.00)	0 (0.00)	0 (0.00)	0 (0.00)
20-25	23 (88.46)	0 (0.00)	3 (11.54)	26 (37.68)
25-30	37 (97.37)	1 (2.63)	0 (0.00)	38 (55.07)
>30	5 (100.00)	0 (0.00)	0 (0.00)	5 (7.25)
Total	65 (94.20)	1 (1.45)	3 (4.35)	69 (100.00)

As presented in Table 3, the most frequently reported clinical presentation was passage of grape-like vesicles, observed in 27 (39.13%) patients, followed closely by bleeding per vaginum in 26 (37.68%). Hyperemesis gravidarum was reported in 7 (10.14%) patients, amenorrhea in 5 (7.25%), and pain in 4 (5.80%). Among the 26 patients presenting with bleeding per vaginum, 22 (84.62%) had hydatidiform mole, 1 (3.85%) had invasive mole, and 3 (11.54%) had choriocarcinoma. All patients presenting with amenorrhea, pain, hyperemesis gravidarum, or passage of grape-like vesicles had hydatidiform mole. The association between clinical presentation and GTD subtype was not statistically significant according to the Fisher-Freeman-Halton exact test (p = 0.423).

**Table 3:** Clinical Presentation According to Gestational Trophoblastic Disease Subtype (N = 69) Fisher-Freeman-Halton exact test: p = 0.423

Clinical presentation	Hydatidiform mole, number (%)	Invasive mole, number (%)	Choriocarcinoma, number (%)	Total, number (%)
Bleeding per vaginum	22 (84.62)	1 (3.85)	3 (11.54)	26 (37.68)
Amenorrhea	5 (100.00)	0 (0.00)	0 (0.00)	5 (7.25)
Pain	4 (100.00)	0 (0.00)	0 (0.00)	4 (5.80)
Hyperemesis gravidarum	7 (100.00)	0 (0.00)	0 (0.00)	7 (10.14)
Passage of grape-like vesicles	27 (100.00)	0 (0.00)	0 (0.00)	27 (39.13)
Total	65 (94.20)	1 (1.45)	3 (4.35)	69 (100.00)

Table 4 shows the histopathological distribution of the study cases. Complete mole was the predominant histopathological diagnosis, identified in 60 (86.96%) patients. Partial mole was diagnosed in 8 (11.59%) patients, while invasive mole was detected in 1 (1.45%) patient. Thus, complete mole constituted the overwhelming majority of histopathologically diagnosed cases in the study population.

**Table 4:** Distribution According to Histopathological Examination (N = 69)

Histopathological diagnosis	Number	%
Complete mole	60	86.96
Partial mole	8	11.59
Invasive mole	1	1.45
Total	69	100.00

According to the ultrasonographic findings presented in Table 5, complete mole was the most common ultrasonographic diagnosis and was observed in 55 (79.71%) patients. Partial mole was identified in 13 (18.84%) patients, whereas invasive mole was detected in 1 (1.45%) patient. Therefore, ultrasonography most frequently demonstrated features consistent with complete hydatidiform mole.

**Table 5:** Distribution According to Ultrasonographic Findings (N = 69)

Ultrasonographic diagnosis	Number	%
Complete mole	55	79.71
Partial mole	13	18.84
Invasive mole	1	1.45
Total	69	100.00

Treatment outcomes are summarized in Table 6. Complete resolution or remission was achieved in 62 (89.86%) of the 69 patients, indicating a favorable outcome in the majority of cases. Persistent gestational trophoblastic neoplasia, excluding invasive mole, was observed in 6 (8.70%) patients, while 1 (1.45%) patient had invasive mole. Overall, nearly 9 out of every 10 patients achieved complete resolution or remission following treatment and follow-up.

**Table 6:** Treatment Outcomes of Patients With Gestational Trophoblastic Disease (N = 69) GTN: gestational trophoblastic neoplasia

Treatment outcome	Number	%
Complete resolution/remission	62	89.86
Persistent GTN excluding invasive mole	6	8.70
Invasive mole	1	1.45
Total	69	100.00

Table 7 presents the distribution of patients according to pre-evacuation serum β-hCG levels. A markedly elevated serum β-hCG level of >100,000 mIU/mL was observed in 60 (86.96%) patients. Nine (13.04%) patients had serum β-hCG levels between 15,000 and 100,000 mIU/mL, while none had a level below 15,000 mIU/mL. These findings indicate that the majority of patients presented with substantially elevated pre-evacuation serum β-hCG concentrations.

**Table 7:** Distribution According to Pre-evacuation Serum β-hCG Levels (N = 69) β-hCG: β-human chorionic gonadotropin

Pre-evacuation serum β-hCG level (mIU/mL)	Number	%
<15,000	0	0.00
15,000-100,000	9	13.04
>100,000	60	86.96
Total	69	100.00

## Discussion

In the present study, the largest proportion of women with gestational trophoblastic disease (GTD) belonged to the 25-30-year age group, comprising 38 (55.07%) patients, followed by 26 (37.68%) patients aged 20-25 years and 5 (7.25%) patients aged >30 years. No patient was younger than 20 years. Regarding parity, 54 (78.26%) participants had a parity of ≥2, whereas 15 (21.74%) had a parity of <2. Bhat and Nagarsenkar (2025), in a study of 42 women with molar pregnancy in Goa, reported a mean age of 25.78 ± 6.1 years; 40.5% were aged 26-30 years, 23.8% were aged 21-25 years, 23.8% were aged 15-20 years, and 11.9% were older than 30 years [10]. They also reported that 61.9% of the women were multiparous, including 57.1% multiparous and 4.8% grand multiparous, compared with 78.26% having parity ≥2 in the present study. Thus, both studies demonstrated a concentration of GTD cases within the main reproductive age groups, although a greater proportion of women in the present study had higher parity. As the present study did not include a control group, these descriptive distributions should not be interpreted as evidence that a particular age group or higher parity independently increases the risk of GTD.

Regarding educational status, 41 (59.42%) women had no formal education, while 14 (20.29%) had primary education, 11 (15.94%) had secondary education, and only 3 (4.35%) had tertiary education. Housewives accounted for 38 (55.07%) participants, while 31 (44.93%) were working women. Islam et al. (2024), in a study of 50 cases of molar pregnancy in Bangladesh, reported that 58% of participants were well educated, 20% were literate, and 22% were illiterate. They also reported that 76% of the participants were housewives, 10% were government employees, 8% worked in non-governmental organizations, and 6% were students. The present study therefore had a larger proportion of women without formal education and a lower proportion of housewives. In terms of religion, 43 (62.32%) participants were Muslim, 19 (27.54%) were Hindu, and 7 (10.14%) belonged to other religions. These proportions most likely reflect the demographic characteristics and referral population served by the hospital and should not be interpreted as demonstrating an association between religion and GTD in the absence of an appropriate comparison population [11].

Hydatidiform mole was the predominant GTD subtype in the present study, accounting for 65 (94.20%) of the 69 cases. Choriocarcinoma was observed in 3 (4.35%) patients and invasive mole in 1 (1.45%), while no case of placental site trophoblastic tumor was identified. Most GTD cases occurred among women aged 25-30 years, followed by those aged 20-25 years. All three cases of choriocarcinoma occurred in the 20-25-year age group, whereas the single invasive mole occurred in the 25-30-year age group. However, the association between age group and GTD subtype was not statistically significant according to the Fisher-Freeman-Halton exact test (p = 0.186). Jagtap et al. (2017), in a clinicopathological study of 77 cases of GTD, similarly reported hydatidiform mole as the predominant diagnosis, accounting for 74 (96.1%) cases. Invasive mole, choriocarcinoma, and placental site trophoblastic tumor each accounted for 1 (1.3%) case. Thus, the proportion of hydatidiform mole reported by Jagtap et al. was comparable to the 94.20% observed in the present study. Jagtap et al. also reported that 57.1% of cases occurred in the 20-25-year age group and 32.5% in the 25-30-year age group, whereas the present study showed a greater concentration in the 25-30-year age group [12]. These variations may be attributable to regional differences in reproductive patterns, referral characteristics, duration of data collection, and the relatively small number of malignant GTD cases.

The most frequent clinical presentation in the present study was passage of grape-like vesicles, reported in 27 (39.13%) patients, followed by bleeding per vaginum in 26 (37.68%), hyperemesis gravidarum in 7 (10.14%), amenorrhea in 5 (7.25%), and pain in 4 (5.80%). All three patients with choriocarcinoma and the single patient with invasive mole presented with bleeding per vaginum. Nevertheless, the overall association between clinical presentation and GTD subtype was not statistically significant according to the Fisher-Freeman-Halton exact test (p = 0.423). Ocheke et al. (2011), in a study of 25 case records from Nigeria, reported vaginal bleeding in 92% of patients and passage of vesicles in 60% [13]. These proportions were considerably higher than the corresponding values of 37.68% and 39.13% observed in the present study. This difference may partly be related to the method of recording clinical presentation, as the categories in the present study represented the principal presenting complaint and summed to the total number of patients, whereas patients in the Nigerian study may have had multiple simultaneous clinical manifestations. Earlier diagnosis through ultrasonography and serum β-hCG testing, differences in referral delay, and retrospective under-documentation of secondary symptoms may also have contributed. Nevertheless, both studies highlight vaginal bleeding and passage of vesicular tissue as important clinical manifestations that should prompt evaluation for GTD.

Histopathological examination demonstrated complete mole in 60 (86.96%) cases, partial mole in 8 (11.59%), and invasive mole in 1 (1.45%). Complete mole was therefore the predominant histopathological diagnosis. Lakra et al. (2017), in a study of 38 women with GTD at a rural tertiary center in Haryana, reported molar pregnancy in 33 (86.8%) patients and gestational trophoblastic neoplasia in 5 (13.2%). Among the 33 molar pregnancies, 27 (81.8%) were complete moles and 6 (18.2%) were partial moles. Complete mole was therefore the predominant histopathological subtype in both studies, although its proportion was somewhat higher in the present study, whereas partial mole was less frequent. Differences in gestational age at evacuation, histopathological interpretation, diagnostic criteria, availability of p57 immunohistochemistry or ploidy analysis, and referral patterns may account for this variation. Because early complete and partial moles can demonstrate overlapping morphological features, histopathological findings should be interpreted in conjunction with ultrasonographic, clinical, and β-hCG findings, particularly when ancillary diagnostic techniques are unavailable [14].

Ultrasonography demonstrated features suggestive of complete mole in 55 (79.71%) patients, partial mole in 13 (18.84%), and invasive mole in 1 (1.45%). Complete mole was therefore also the predominant ultrasonographic finding. Fowler et al. (2006) evaluated 1,053 suspected cases and reported that, following expert histopathological review, 859 were confirmed as complete or partial hydatidiform moles. Pre-evacuation ultrasonography suggested a molar pregnancy in only 378 (44%) of these confirmed cases. Importantly, ultrasonography detected 200 of 253 complete moles (79%) but only 178 of 606 partial moles (29%), demonstrating substantially better detection of complete than partial moles. The 79.71% proportion of complete-mole diagnoses on ultrasonography in the present study is numerically similar to the 79% detection rate for complete mole reported by Fowler et al.; however, these values represent different measures. The present value describes the distribution of ultrasonographic diagnoses, whereas Fowler et al. reported the detection rate among histologically confirmed cases. In the present study, histopathological examination identified 60 complete and 8 partial moles, whereas ultrasonography identified 55 complete and 13 partial moles. This difference suggests that some lesions initially considered partial moles on imaging may have subsequently been classified differently following tissue examination. However, patient-level cross-tabulation of ultrasonographic and histopathological findings would be necessary to calculate diagnostic sensitivity, specificity, predictive values, or agreement [15].

Treatment outcomes were generally favorable, with complete resolution or remission achieved in 62 (89.86%) patients. Persistent gestational trophoblastic neoplasia, excluding invasive mole, was observed in 6 (8.70%) patients, while 1 (1.45%) patient had invasive mole. Thus, 7 (10.14%) patients had persistent or invasive disease requiring further assessment or treatment. Fatima et al. (2011), in a prospective study of 85 molar pregnancies in Quetta, Pakistan, reported that none of the patients developed persistent trophoblastic disease, invasive mole, or choriocarcinoma during a mean follow-up period of 5.7 months, with a range of 1-24 months. Their observed remission rate was therefore higher than the 89.86% recorded in the present study. This difference may be related to variation in case selection because the present study included different forms of GTD, including invasive and malignant disease, whereas Fatima et al. primarily evaluated molar pregnancies. Differences in baseline disease severity, proportions of complete and partial mole, pretreatment β-hCG concentrations, duration and completeness of follow-up, and diagnostic criteria for persistent GTN may also have contributed. Nevertheless, the high remission rate observed in the present study emphasizes the importance of timely diagnosis, appropriate treatment, and post-treatment surveillance with serial β-hCG measurements [16].

Pre-evacuation serum β-hCG levels were markedly elevated in the study population. Sixty (86.96%) patients had concentrations >100,000 mIU/mL, while 9 (13.04%) had concentrations between 15,000 and 100,000 mIU/mL; none had concentrations below 15,000 mIU/mL. Balsarkar et al. (2024), in a prospective study of 67 women undergoing uterine evacuation for missed or incomplete abortion, identified 5 (7.5%) histologically confirmed molar pregnancies. Only 4 (6.0%) women in their entire cohort had β-hCG levels >100,000 mIU/mL; however, all 4 had molar pregnancy. Thus, 4 of the 5 molar pregnancies (80%) were associated with β-hCG values above 100,000 mIU/mL, which is comparable to the 86.96% observed in the present GTD cohort. The association between β-hCG >100,000 mIU/mL and molar gestation was highly significant in their study (p < 0.0001). These findings support the clinical importance of markedly elevated β-hCG concentrations in the evaluation of suspected GTD. However, serum β-hCG should not be used in isolation to determine GTD subtype and should instead be interpreted together with clinical presentation, ultrasonography, histopathological examination, uterine size, and serial post-evacuation trends. Persistently elevated or rising β-hCG after treatment remains particularly important for identifying persistent or neoplastic trophoblastic disease [17].

Limitations

The present study was limited by its retrospective, single-center design and relatively small sample size of 69 patients, which may limit the generalizability of the findings. Reliance on hospital records may have resulted in incomplete documentation of some clinical variables, while the absence of a control group precluded assessment of independent risk factors for GTD. In addition, the limited number of malignant and persistent GTD cases restricted the ability to perform multivariable analyses.

## Conclusions

Hydatidiform mole was the predominant form of gestational trophoblastic disease, with complete mole representing the most common histopathological and ultrasonographic finding. Most patients achieved complete resolution or remission following treatment, although a small proportion developed persistent or invasive disease. Markedly elevated pre-evacuation serum β-hCG levels were common, emphasizing the importance of integrated clinical assessment, ultrasonography, histopathological confirmation, and serial β-hCG surveillance for effective diagnosis, treatment, and follow-up.
